# Persistent distention of colon damages interstitial cells of Cajal through Ca^2+^‐ERK‐AP‐1‐*miR‐34c*‐SCF deregulation

**DOI:** 10.1111/jcmm.13108

**Published:** 2017-06-04

**Authors:** Shu Yang, Fang Dong, Dandan Li, Haimei Sun, Bo Wu, Tingyi Sun, Yaxi Wang, Ping Shen, Fengqing Ji, Deshan Zhou

**Affiliations:** ^1^ Department of Histology and Embryology School of Basic Medical Sciences Capital Medical University Beijing China; ^2^ Beijing Key Laboratory of Cancer Invasion and Metastasis Research Beijing China

**Keywords:** AP‐1/c‐Jun, distention, gastrointestinal motility disorder, interstitial cell of Cajal, *miR‐34c*, stem cell factor, stretch

## Abstract

Gastrointestinal motility disorders (GMDs) are attributed to loss of interstitial cells of Cajal (ICC), whose survival and function are deeply dependent on the activation of KIT/SCF signalling. Based on the facts that gastrointestinal distention is common in GMD patients and SCF produced by smooth muscle cells (SMCs) is usually decreased before ICC loss, we considered a possible contribution of persistent gastrointestinal distention/stretch to SCF deficiency. In this study, chronic colonic distention mouse model, diabetic gastrointestinal paresis mouse model, cultured mouse colonic SMCs and colon specimens from Hirschsprung's disease patients were used. The results showed that SCF was clearly decreased in distent colon of mice and patients, and microRNA array and real‐time PCR indicated a concomitant increase of *miR‐34c* in distent colon. A negative regulation of *miR‐34c* on SCF expression was confirmed by luciferase reporter assays together with knock‐down and overexpression of *miR‐34c* in cultured colonic SMCs. Using EMSA and ChIP assays, we further consolidated that in response to persistent stretch, the transcription factor AP‐1/c‐Jun was highly activated in colonic SMCs and significantly promoted *miR‐34c* transcription by binding to *miR‐34c* promoter. Knock‐down or overexpression of AP‐1/c‐Jun in cultured colonic SMCs leads to down‐ or up‐regulation of *miR‐34c*, respectively. In addition, the activation of AP‐1/c‐Jun was through ERK1/2 signalling provoked by Ca^2+^ overload in colonic SMCs that were subject to persistent stretch. In conclusion, our data demonstrated that persistent distention/stretch on colonic SMCs could suppress SCF production probably through Ca^2+^‐ERK‐AP‐1‐*miR‐34c* deregulation, resulting in ICC loss or impairment and GMD progress.

## Introduction

GMDs, such as diabetic gastroparesis, slow transit constipation, Hirschsprung's disease (HD) and chronic intestinal pseudo‐obstruction, show increasing prevalence. In addition to the common manifestations, *e.g*. abdominal distension, abdominal pain, nausea, vomiting and constipation, GMDs could cause life‐threatening complications including intestinal ischaemia, necrosis and intestinal neoplasm. Efforts to explore the pathogenesis of GMDs have been ongoing for years, and piles of documents denoted a significance of the loss of ICC and the disruption of their networks in the gastrointestinal (GI) tract of GMD patients 1, 2, 3.

ICC, located in the smooth muscle layers of the GI tract and forming networks with each other *via* their cell processes, act as pacemakers for the GI movements and integrate excitatory and inhibitory neurotransmission with slow‐wave activity 4, 5. Therefore, damages to ICC are closely responsible for the development of GMDs. It is well known that the survival, proliferation and function of ICC deeply depend on the activation of the membrane receptor KIT by its natural unique ligand, stem cell factor (SCF) that mainly produced by the GI SMCs and enteric neurons 6. Evidence showed that SCF mutant (*Sl/Sld*) mice had a dramatic decrease in ICC and disrupted ICC networks 7. Lin *et al*. 8 elucidated that a depletion of ICC in the colon of diabetic mice was attributed to a deficiency in SCF production. Furthermore, exogenous SCF improved ICC restoration after blocking KIT signalling pathway 9. Significantly reduced SCF in diabetic mice was a pivotal contributor to ICC loss and consequent gastroparesis 10. Consistently, in our diabetic gastrointestinal paresis (DGIP) mice, SCF production was evidently decreased prior to ICC loss. These results also highlighted the key role of SCF in the maintenance of ICC in the GI tract and indicated that reduced SCF would lead to GMDs. However, the underlying mechanism of SCF decrease during the development of GMDs remains incompletely elucidated.

The non‐coding microRNAs have been attractive because they post‐transcriptionally down‐regulate target mRNAs that initiate or facilitate the development of multiple diseases. Recent studies revealed that abnormal microRNA profile in SMCs and serum had a direct bearing on GMDs 11, 12. But up to date, there was no evidence showing whether and which microRNA(s) could be responsible for the down‐regulation of SCF in SMCs so as for GMDs. Moreover, what triggered the potential alteration of microRNAs‐SCF is still unclear. Based on the literatures, we noticed that GI distention is a common pathological feature in GMD patients 13, 14. Previous studies reported that stretch could arouse intracellular signalling cascade by activating membrane sensors, *e.g*. integrins, calcium (Ca^2+^) channel, in airway and vascular SMCs 15, 16. Recently, we found a Ca^2+^ overload in stretched colonic SMCs and consequent activation of MAPK signalling *in vivo* and *in vitro* 17. Yet the exact molecules activated by persistent stretch that could induce alterations of microRNAs‐SCF have not been uncovered.

Therefore, the present study concentrated on the downstream molecules induced by persistent stretch using chronic colonic distention mouse model, DGIP mouse model, cultured mouse colonic SMCs and colon specimens from HD patients who are well acknowledged to have severe ICC dysfunction 2, to better understand the SCF deficiency‐related GMDs. We expect our results could be beneficial for finding specific target molecules in preventing or ameliorating GMDs in clinic.

## Materials and methods

### Establishment of mouse models

Male BALB/c mice (6 week old, 22–26 g) were purchased from the Animal Center of Capital Medical University. All mice were maintained in a temperature‐controlled room (23 ± 1°C) with a constant 12‐hrs light/dark cycle. Food and water were available *ad libitum*. All experimental procedures were approved by the Institutional Animal Care Committee from Capital Medical University, Beijing China.

Thirty mice were used to establish chronic colonic distention model by incomplete colon obstruction operation, as previously described by Won *et al*. 18. Briefly, mouse was anesthetized by an intra‐peritoneal injection of 4% chloral hydrate (0.01 ml/g). About 1‐cm incision was made in lower right abdomen, and proximal colon was exposed. A sterile plastic tube (1 mm in diameter) was tied with colon by a 3–0 silk suture at a position of 2 cm away from caecum. The tube was then removed to achieve incomplete colon obstruction. Another 10 sham‐operated mice underwent the same procedure but without incomplete colon obstruction treatment.

Thirty mice were used to establish DGIP model. Each mouse received a single intra‐peritoneal injection of alloxan monohydrate (200 mg/kg, Sigma‐Aldrich, St. Louis, MO, USA). Five littermates receiving the same dose of normal saline (NS) were set as controls. Fasting blood glucose was measured by the ACCU‐CHEK Active Complete blood glucose monitor (Roche, Grenzach‐Wyhlen, Germany) 72 hrs later. The mice with blood glucose ≥11.1 mmol/l were considered as type 1 diabetes mice. Eight weeks after alloxan administration, the mice were killed by an overdose of 4% chloral hydrate (0.02 ml/g) following the GI propulsive distance rate (PDR) testing.

### Gastrointestinal PDR testing

Each diabetes and control mouse received an oral administration of 0.2 ml carbonic ink after fasting for 12 hrs. Thirty min later, the mice were killed and GI length (L) and ink propulsion distance (D) were measured. The PDR was calculated according to: PDR = D/L × 100%.

### Cell culture and stretch exertion

Mouse colonic SMCs were obtained from ROCHEN Inc. (Shanghai, China) and cultured in Dulbecco's modified Eagle medium (DMEM/F12; Life Technologies, Carlsbad, CA, USA) with 15% foetal bovine serum (FBS, Life Technologies) at 37°C with 95% air and 5% CO_2_. SMCs (10^5^ cells/well) were seeded into 6‐well Bioflex plates (Flexcell, Burlington, NC, USA) coated with collagen I. When reaching 90% confluence, SMCs were cultured in serum‐free medium to induce quiescence for 24 hrs. Then SMCs were subject to 16% strain of persistent stretch for 12 hrs in a computer‐controlled stress unit (Flexcell). Cells cultured in static condition were set as controls.

### Human colon specimens

Human distent colon specimens and adjacent normal specimens were obtained from 10 HD patients aged 0–3 month in Beijing Children's Hospital, Capital Medical University (Beijing, China). All procedures were performed with written informed consents by the guardians according to the Declaration of Helsinki and the research was approved by the Ethics Committee of Capital Medical University.

### MicroRNA array

Total RNA was extracted from the colonic smooth muscles with TRIzol reagent (Life Technologies), and the microRNA expression profile of the samples was analysed by KangChen Bio‐tech (Shanghai, China) using the Exiqon microRNA Microarray System (Exiqon, Vedbaek, Denmark). The expression of individual microRNA was further confirmed by real‐time quantitative PCR.

### Real‐time quantitative PCR

Total microRNA was extracted from the colonic smooth muscle tissues and cultured colonic SMCs using miRNApure Mini Kit (CWBiotech, Beijing, China) according to the manufacturer's instruction. Reverse transcription was performed using Taqman microRNA RT Kit (Life Technologies) and Taqman microRNA Assay with specific stem‐loop primers (Life Technologies). Real‐time PCR was performed with Taqman Universal Master Mix II (Life Technologies) and Taqman microRNA Assay (Life Technologies). The reactions were incubated at 95°C for 10 min., followed by 40 cycles of 95°C for 15 sec. and 60°C for 1 min. in the ABI 7500 real‐time PCR system (Applied Biosystems, Foster city, CA, USA). Results were normalized to the internal control, *RNU6B*.

Total RNA was extracted from the colonic smooth muscle tissues and cultured colonic SMCs with TRIzol reagent. Reverse transcription was performed using High‐Capacity RNA‐to‐cDNA Kit (Life Technologies). Real‐time PCR was performed using SYBR Green PCR Master Mix (Life Technologies) in the ABI 7500 real‐time PCR system. The following primers were used: *SCF* (Forward CAGAGTCAGTGTCACAAAACCATT, Reverse TTGGCCTTCCTATTACTGCTAC TG); *GAPDH* (Forward AGAAGGCTGGGGCTCATTTG, Reverse AGGGGCCATC CACAGTCTTC). The reactions were incubated at 95°C for 10 min., followed by 40 cycles of 95°C for 15 sec. and 60°C for 1 min. Results were normalized to the internal control, *GAPDH*.

All reverse transcription reactions included no‐template controls, and all PCR were run in triplicate. Relative gene expression was determined using the comparative C_T_ (2^−▵▵CT^) method.

### Western blotting

Colonic smooth muscle tissues and cultured colonic SMCs were lysed in RIPA buffer (Applygen, Beijing, China) with 1% phosphatase inhibitor cocktail (Sigma‐Aldrich), 1% protease inhibitor cocktail (Sigma‐Aldrich) and 1% phenylmethanesulphonyl fluoride (PMSF, Solarbio, Beijing, China). Proteins were separated on 10% SDS‐PAGE gel and transferred onto PVDF membrane (Merk‐Millipore, Temecula, CA, USA), followed by blockage with 5% non‐fat milk or bovine serum albumin (BSA, Sigma‐Aldrich) for 1 hr. The membranes were incubated with individual primary antibody (Table S1) at 4°C overnight. Then the membranes were incubated with corresponding HRP‐conjugated secondary antibody (Table S1) at 25°C for 1 hr. The bands were detected with ECL chemiluminescence (Thermo Scientific, Waltham, MA, USA) and viewed in Fusion FX Vilber Lourmat (France).

### Immunofluorescence staining

Whole mount preparations were obtained according to our previous study 19. Cryosections (8 μm thick) were cut with a cryostat (Leica CM3050S, Germany). After fixed with 4% paraformaldehyde or acetone for 30 min., the samples were blocked with 1% BSA for 1 hr, followed by the incubations with individual primary antibody (Table S1) at 4°C overnight and Cy3‐conjugated secondary antibody (Table S1) at 25°C for 1 hr. The samples were mounted with fluorescent mounting medium containing DAPI (ZSGB Bio, Beijing, China) and visualized with the fluorescence microscope (Nikon, Ni, Japan) or the laser scanning confocal microscope (TCS SP5; Leica, Germany). Specificity was verified by omitting the primary antibody and by pre‐absorption with appropriate blocking peptide.

### Bioinformatics

The putative microRNAs that could be able to bind to the 3′‐untranlational region (UTR) of *SCF* mRNA were predicted with the TargetScanMouse, MiRanda and miRBase programs. The putative promoter sequence of *miR‐34c* was retrieved from the UCSC Genome Brower. Prediction of transcription factors for *miR‐34c* was conducted using the TFSearch.

### Overexpression and knock‐down of *miR‐34c*

The full length of *pre‐miR‐34c* was chemically synthesized by GeneChem and introduced into the GV217 lentiviral vector (GeneChem, Shanghai, China) in the unique EcoRI site to construct the lentivirus encoding *miR‐34c* (GV217‐*miR‐34c*). The specific inhibitor of *miR‐34c* was constructed by cloning the complementary nucleotides of *miR‐34c* into the GV280 lentiviral vector (GV280‐inhibitor, GeneChem) between the AgeI and EcoRI sites. The mouse colonic SMCs were seeded in a 6‐well plate at a density of 5 × 10^4^ cells/well and were infected with lenti‐*miR‐34c* or its inhibitor when the cells reached 30% confluence. Three days later, the efficiency of infection was evaluated by observing EGFP expression with the fluorescence microscope (Nikon, Ni; Fig. S1A). Overexpression and knock‐down of *miR‐34c* were further confirmed by real‐time PCR.

### Plasmid construction and dual‐luciferase reporter assay

The 3′‐UTR sequences of *SCF* mRNA were chemically synthesized and introduced into the GV306 luciferase reporter vector (GeneChem) at the unique XbaI site to generate GV306‐*SCF*. The seed regions of *miR‐34c* in the *SCF* 3′‐UTR were mutated to construct GV306‐*SCF*‐MUT. The GV262‐*miR‐34c* construct was created by introducing the *miR‐34c* sequence into the GV262 vector at the XhoI/EcoRI sites (GeneChem). A construct containing an unrelated microRNA was used as a negative control (GV262‐control).

To construct the *miR‐34c* promoter–luciferase reporter plasmid (GV238 promoter), 1‐kb region of the *miR‐34c* promoter was chemically synthesized by GeneChem and subsequently cloned into the KpnI and XhoI sites of the GV238‐basic plasmid (GeneChem). The *miR‐34c* promoter reporter plasmid containing site‐specific mutagenesis for c‐Jun binding site was generated using QuikChange Lightning Site‐Directed Mutagenesis Kit (Agilent Technologies, Santa Clara, CA, USA) and cloned into the GV238‐basic plasmid (GeneChem). The c‐Jun overexpression plasmid was chemically synthesized and cloned into the XhoI and KpnI sites of the GV230 vector (GeneChem).

The transfections of 293T cells were performed using Lipofectamine 2000 (Life Technologies), according to the manufacturer's instruction. Twenty‐four hours later, the *Firefly* and *Renilla* luciferase activities were measured by Dual‐Luciferase Assay System (Promega, Madison, WI, USA) in the Multiskan FC (Thermo Scientific), according to the manufacturer's protocol. The ratio of *Firefly* to *Renilla* activity was calculated. Each luciferase assay was performed in 6 times.

### Overexpression and knock‐down of c‐Jun

Mouse colonic SMCs were transfected with the c‐Jun overexpression plasmid (GeneChem) using Lipofectamine 2000 (Life Technologies). Specific siRNA targeting c‐Jun and its negative control siRNA were purchased from Ribobio CO., LTD (Guangzhou, China). siRNAs were transfected into mouse colonic SMCs using riboFECTTM CP Transfection Kit (Ribobio) according to the manufacturer's instruction. Total RNA and total protein were prepared 48 hrs after transfection and used for real‐time PCR and Western blot analysis, respectively.

### Electrophoretic mobility shift assay (EMSA) and super‐shift assay

Nuclear protein extracts of mouse colonic smooth muscle tissues were extracted using nuclear and cytoplasmic extraction reagents (Applygen). EMSA was performed using LightShift Chemiluminescent EMSA Kit (Thermo Scientific) according to the manufacturer's protocol. The 5′‐biotin‐labelled probes, unlabelled probes and unlabelled mutant probes (Table S2) were synthesized by Shanghai Sangon Biological Engineering Technology and Services Company (Shanghai, China). In super‐shift assay, anti‐c‐Jun antibody (Santa Cruz Biotechnology, Santa Cruz, CA, USA) was pre‐incubated with nuclear protein extracts for 5 min. at 25°C. The biotin‐labelled DNA–protein complex was detected using HRP‐conjugated and LightShift chemiluminescent substrate, viewed in Fusion FX Vilber Lourmat.

### Chromatin immunoprecipitation (ChIP) assay

ChIP assays were performed according to the instruction of Magna ChIP™ G Tissue Kit (Merk‐Millipore). Chromatin extracted from mouse colonic smooth muscle tissues was immunoprecipitated for 24 hrs at 4°C using anti‐c‐Jun antibody (Cell Signaling Technology, Danvers, MA, USA). One‐hundredth of the solution collected before adding the antibody was used as an internal control for the amount of input DNA. As a negative control, the antibody or DNA was omitted or replaced with normal rabbit IgG (Cell Signaling Technology). PCR was carried out using 2 × PCR Reagent (TIANGEN, Beijing, China), consisting of 3 min. at 94°C, 32 cycles of 20 sec. at 94°C, 30 sec. at 59°C and 30 sec. at 72°C and 2 min. at 72°C. Primers are shown in Table S3.

### Calcium imaging

Colonic smooth muscle tissues were incubated in Hanks’ solution containing 3 μM Fluo‐4AM and 1% Pluronic F‐127 (Life Technologies) for 40 min. at 37°C in the dark. Fluo‐4 fluorescence was excited at 488 nm using the laser scanning confocal microscope (TCS SP5; Leica). All cells in one field were pooled to obtain the average fluorescence before and after the indicated treatment.

### Statistics

The results were presented as the mean ± S.E.M. and analysed using Student's *t*‐test or one‐way anova with the SPSS 17.0 software (SPSS Inc., Chicago, IL, USA). A value *P* < 0.05 was considered statistically significant.

## Results

### Establishment of mouse models

**Figure 1:**
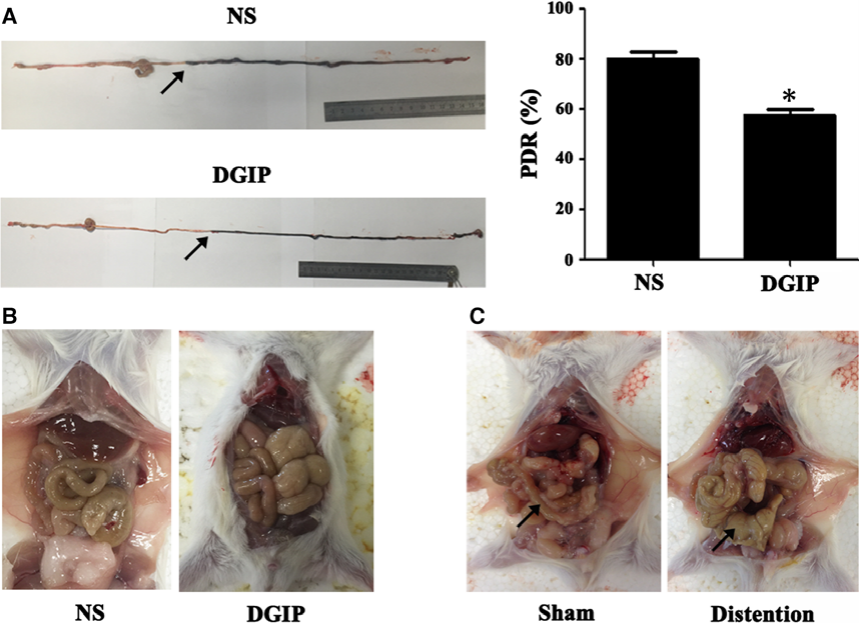
DGIP mice and colonic distention mice were established. (**A**) The left representative figures showed the ink propulsion distances, and the end‐points that the ink travelled were indicated by arrows. The right statistical graph elucidated that the PDR of DGIP mice was significantly decreased by 28.1% compared with NS. (*n* = 5, *P < 0.05) (**B**) The GI tract of DGIP mouse was transparently distent. (**C**) Incomplete colon obstruction operation for 8 days resulted in apparent distention of rostral colon segment. Arrows show the distent colon segment in model mouse and corresponding non‐distent colon segment in sham mouse.

Eight weeks after the alloxan administration, the diabetes mice showed tardy GI propulsive motility, indicated by a significant decrease of 28.1% in PDR (*P* < 0.05; Fig. 1A); therefore, these mice were defined as DGIP mice that, interestingly, had apparent GI distention, one of the main features in GMDs (Fig. 1B). To further confirm the role of GI distension in GMDs, we established a colonic distention mouse model by keeping incomplete colon obstruction for 8 days. As expected, the rostral colon segment to the obstruction site was apparently distent, whereas the corresponding colon segment from the sham‐operated mice was normal (Fig. 1C).

### ICC and SCF were reduced in distent colon

**Figure 2:**
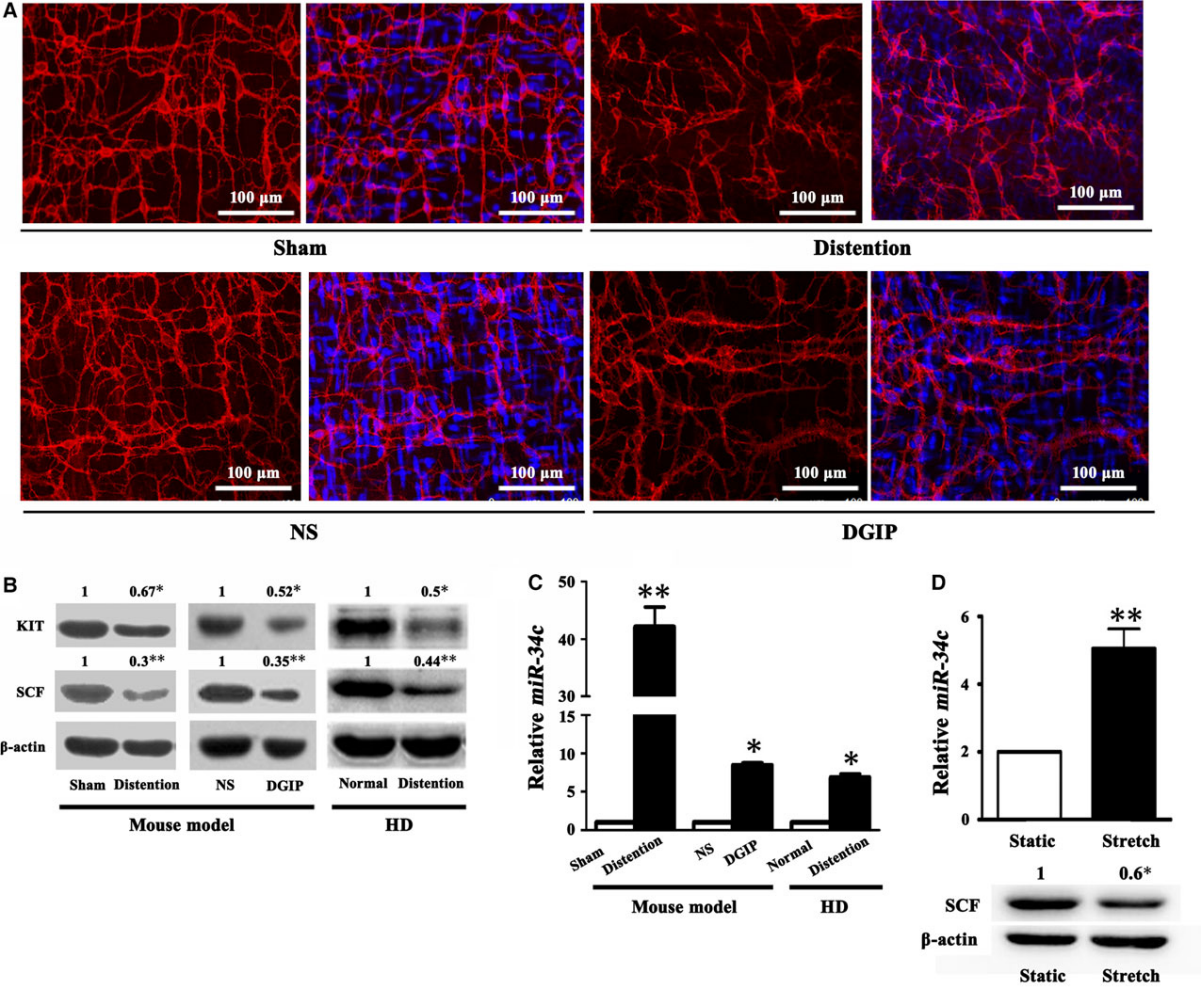
ICC and SCF were reduced, whereas *miR‐34c* was increased in distent colon. (**A**) Within the colonic wall of the colonic distention and DGIP mouse models, KIT‐labelled ICC were apparently decreased and their cellular networks became sparse when compared to the corresponding controls. (**B**) The expressions of KIT and SCF in smooth muscles obtained from the colonic distention mice, DGIP mice and HD patients were significantly decreased compared to the corresponding controls. (*n* = 5 or 10; *P < 0.05; **P < 0.01) (**C**) On the contrary, *miR‐34c* was clearly increased in the distent colonic smooth muscles of the mouse models and HD patients. (*n* = 5 or 10; *P < 0.05; **P < 0.01) (**D**) In the cultured mouse colonic SMCs that were subject to persistent stretch for 12 hrs, *miR‐34c* was significantly elevated, whereas SCF was reduced. (*n* = 5; *P < 0.05; **P < 0.01)

In both colonic distention mice and DGIP mice, ICC were decreased with impaired cellular networks along with hypo‐expressed KIT, when compared with the sham and NS mice, respectively (*P* < 0.05; Fig. 2A and B). Likewise, SCF production in smooth muscles of the colonic distention mice and DGIP mice was significantly decreased (*P* < 0.01; Fig. 2B). Investigations on the specimens from the patients with HD validated the findings in the mouse models, showing that KIT and SCF were attenuated in the distent colon segment compared with the adjacent normal colon segment (*P* < 0.05 or 0.01; Fig. 2B). Collectively, these results clearly indicated that the KIT/SCF signalling was critically restrained in the distent GI tract, which was related to the ICC loss and torpid PDR.

### *miR‐34c* was elevated in distent colon and stretched colonic SMCs

As the SCF deficiency was prior to the ICC loss, we paid attention to the reasons for the SCF deficiency in the colonic distention state, in which microRNAs were highlighted. First, we performed microRNA array to screen out significantly up‐regulated microRNAs in the distent mouse colonic smooth muscles. From the screened out 37 microRNAs, we picked up *miR‐34c* as the candidate because it was supposed to target *SCF* mRNA using bioinformatic method (Fig. S1B). Real‐time PCR results verified that *miR‐34c* was evidently increased in the distent colonic smooth muscles of the mouse models and HD patients (*P* < 0.05 or 0.01; Fig. 2C). To exclude potential interference by inflammation and ischaemia during the incomplete colon obstruction operation, we further carried out *in vitro* persistent stretch for 12 hrs on the cultured mouse colonic SMCs and the results showed that *miR‐34c* was significantly elevated (*P* < 0.01), whereas SCF was reduced (*P* < 0.05) (Fig. 2D).

### Increased *miR‐34c* by persistent stretch down‐regulated SCF expression

**Figure 3:**
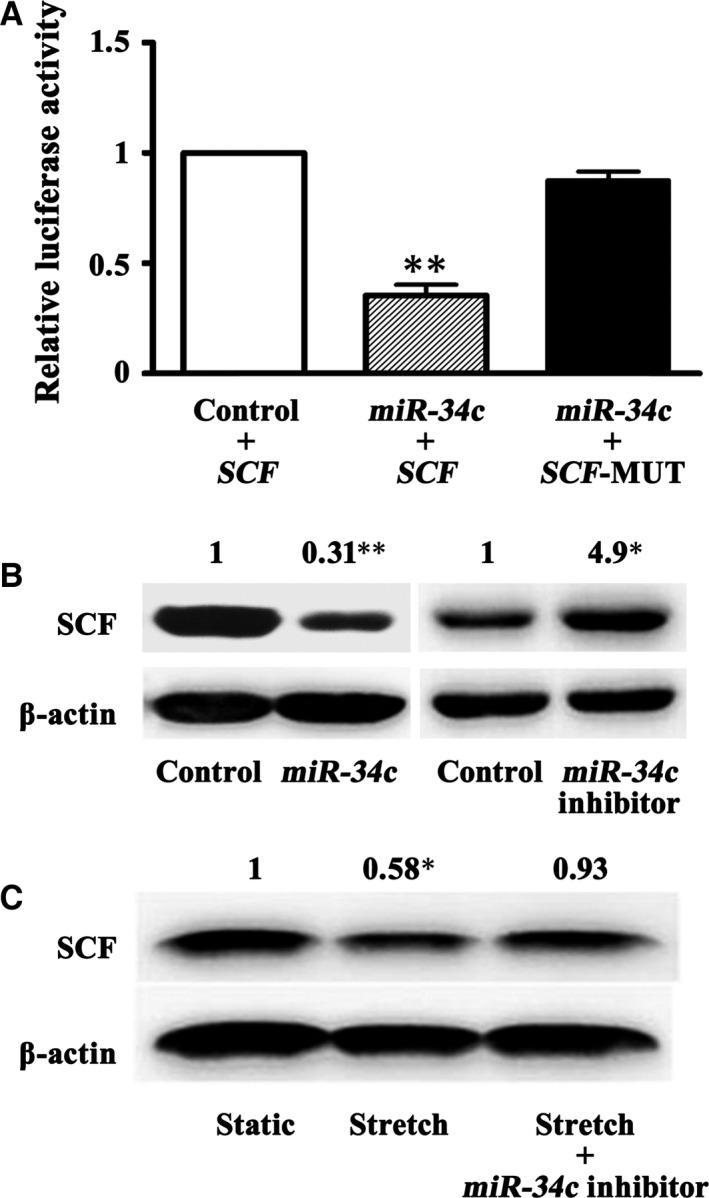
Stretch‐induced *miR‐34c* down‐regulated SCF. (**A**) The luciferase activity was significantly decreased in 293T cells that co‐transfected with plasmids of *miR‐34c* and wild‐type *SCF* 3′‐UTR, whereas co‐transfection with plasmids of *miR‐34c* and mutant *SCF* 3′‐UTR did not alter the luciferase activity. (*n* = 6; **P < 0.01) (**B**) Overexpression of *miR‐34c in vitro* in the mouse colonic SMCs inhibited SCF expression, whereas knock‐down of *miR‐34c* up‐regulated SCF expression. (*n* = 5; *P < 0.05; **P < 0.01) (**C**) Persistent stretch for 12 hrs decreased SCF in the cultured mouse colonic SMCs, which was efficiently abrogated by the additional *miR‐34c* inhibitor. (*n* = 5; *P < 0.05).

The reverse changes in SCF and *miR‐34c* expressions in the distent colonic smooth muscles strongly implied a regulation of *miR‐34c* on SCF expression. Dual‐luciferase reporter assays showed that *miR‐34c* definitely bound to the 3′‐UTR of *SCF* mRNA, indicated by the decreased luciferase activity when 293T cells were co‐transfected with *miR‐34c* and *SCF* 3′‐UTR (*P* < 0.01; Fig. 3A). By over expressing and knocking down endogenous *miR‐34c* in the mouse colonic SMCs, SCF expression was sharply down‐ and up‐regulated, respectively (*P* < 0.05 or 0.01; Fig. 3B). Significantly, the persistent stretch‐induced decrease in SCF in the mouse colonic SMCs was abrogated by the introduction of *miR‐34c* inhibitor (*P* < 0.05; Fig. 3C). These results suggested that the persistent stretch‐induced increase of *miR‐34c* could potently reduce SCF.

### c‐Jun promoted *miR‐34c* transcription under stretched condition

**Figure 4:**
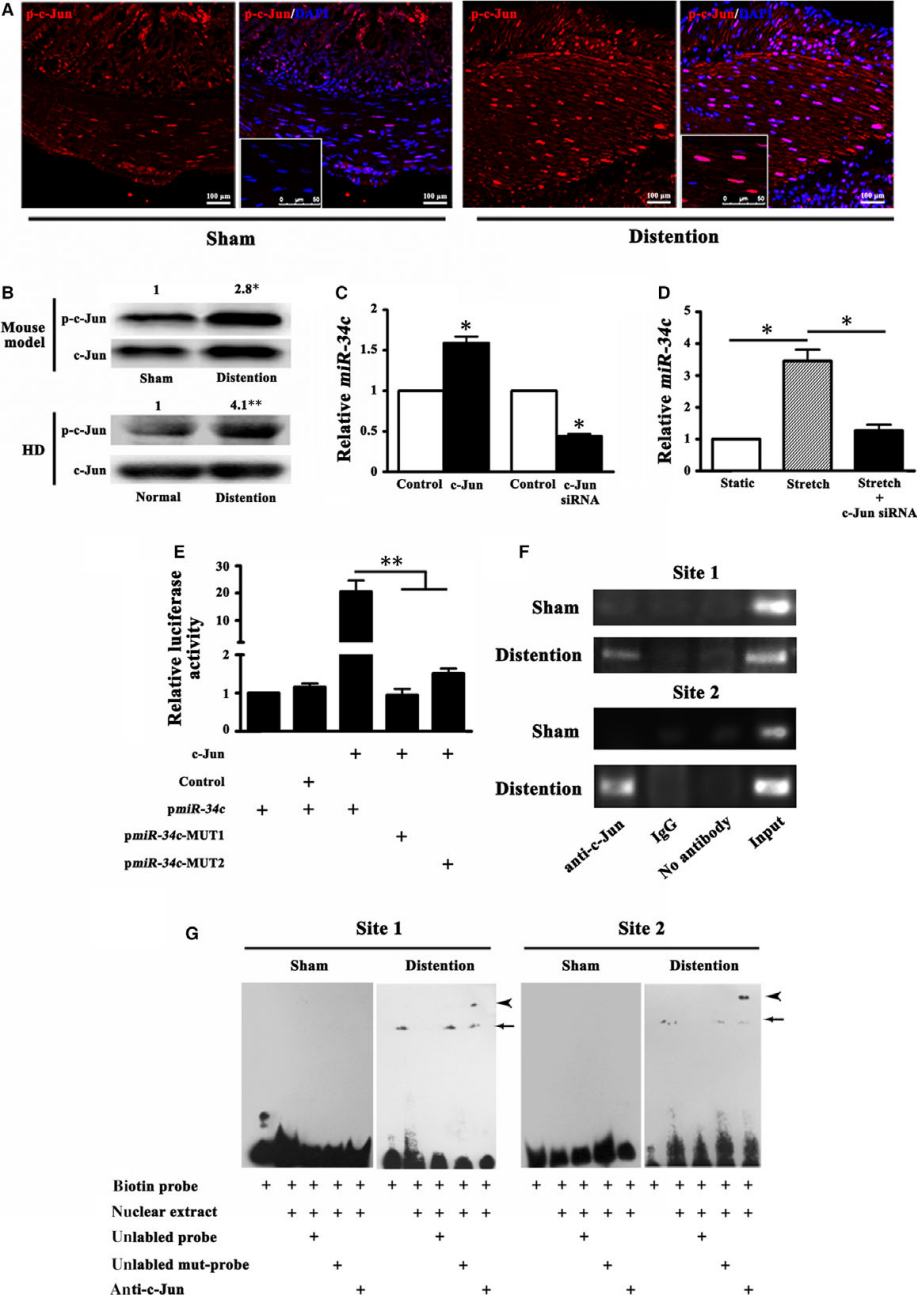
c‐Jun promoted *miR‐34c* transcription under stretched condition. (**A**) In the colonic distention mice, the activation of nuclear factor c‐Jun was much more conspicuous in the nuclei of colonic SMCs. The framed panels showed that the phosphorylated c‐Jun (p‐c‐Jun) was located within the nuclei. (**B**) In the distent colonic smooth muscles of the mouse model and HD patients, the expression of p‐c‐Jun was evidently increased compared to corresponding controls. (*n* = 5 or 10; *P < 0.05; **P < 0.01) (**C**) Overexpression of c‐Jun *in vitro* in the mouse colonic SMCs increased *miR‐34c* expression, whereas knock‐down of c‐Jun by its specific siRNA reduced *miR‐34c* level. (*n* = 5; *P < 0.05) (**D**) The persistent stretch‐induced up‐regulation of *miR‐34c* was overtly counteracted by the knock‐down of c‐Jun in the cultured mouse colonic SMCs. (*n* = 5; *P < 0.05) (**E**) 293T cells were transfected with the wild‐type *miR‐34c* promoter (p*miR‐34c*) or its mutants (p*miR‐34c*‐MUT‐1/2) whose binding site for c‐Jun was mutated separately, in the presence of c‐Jun or its control plasmid. The relative luciferase activity of p*miR‐34c* construct was remarkably increased when co‐transfected with c‐Jun, whereas the luciferase activities of p*miR‐34c*‐MUT‐1 and p*miR‐34c*‐MUT‐2 constructs were lowered even co‐transfected with c‐Jun. (*n* = 6; **P < 0.01) (**F**) ChIP assays were performed on the distent mouse colonic smooth muscles and sham controls. Chromatin was immunoprecipitated with the anti‐c‐Jun antibody. The PCR specific to Site 1 and Site 2 were performed. The bands were clearer after the incubation with anti‐c‐Jun antibody in the distent colonic smooth muscles than those in the sham controls. (*n* = 5) (**G**) Nuclear extract from colonic smooth muscles of the distent and sham control mice were incubated with biotin‐labelled probe corresponding to putative c‐Jun binding Site 1 and Site 2 in the *miR‐34c* promoter. For competition system, 200‐fold excess of unlabelled probe or unlabelled mutational probe was additionally added. For super‐shift assay, anti‐c‐Jun antibody was included. The negative control was used in the absence of nuclear extracts. It clearly showed that c‐Jun specifically bound to the Site 1 and Site 2 within the *miR‐34c* promoter when the colon was distent. The arrows indicate the specific DNA–protein complex and the arrowheads indicate the super‐shift.

Up to date, little is known about the regulation of *miR‐34c* transcription in the colonic SMCs. It was suggested that activator protein‐1 (AP‐1) transcription factor played a crucial role in gene transcription in bladder and vascular SMCs in response to mechanical forces 20, 21. However, whether AP‐1 could also enhance *miR‐34c* transcription in colonic SMCs when stretched was not unveiled. Our results showed that, parallel with the increase of *miR‐34c*, the phosphorylation of c‐Jun, an important subunit of AP‐1, in the nuclei of SMCs was obviously greater in the colonic distention mice and patients with HD than that in their respective controls (*P* < 0.05 or 0.01; Fig. 4A and B). *In vitro* overexpression or knock‐down of c‐Jun in the cultured mouse colonic SMCs caused increase or decrease of *miR‐34c*, respectively (*P* < 0.05; Fig. 4C). Moreover, *miR‐34c* expression was enhanced upon the treatment of 100 nM TPA, a transcription inducer from AP‐1‐driven promoter, in a time‐dependent manner (*P* < 0.05; Fig. S2A). Noticeably, knock‐down of c‐Jun by its specific siRNA counteracted the persistent stretch‐induced increase of *miR‐34c* (*P* < 0.05; Fig. 4D), strongly indicating that c‐Jun was involved in the stretch‐induced *miR‐34c* transcription.

Next, we looked forward to finding out how c‐Jun facilitated *miR‐34c* transcription. Through online transcription factor prediction programs, we found 2 putative binding sites of c‐Jun within the 1‐kb region upstream of the *pre‐miR‐34c*, Site 1—405~−395 bp and Site 2—371~−361 bp (Fig. S2B). We used site‐directed mutagenesis to abolish each binding site and performed luciferase assays. The luciferase activities of p*miR‐34c*‐MUT‐1 and p*miR‐34c*‐MUT‐2 constructs were strikingly decreased by 95.2% (*P* < 0.01) and 92.4% (*P* < 0.01), respectively (Fig. 4E), compared with the wild‐type *miR‐34c* promoter (pmiR‐34c), demonstrating that the *miR‐34c* transcription was deeply dependent on the presence of c‐Jun binding to the promoter.

The binding property of c‐Jun to the *miR‐34c* promoter was validated by ChIP assays on the distent mouse colonic smooth muscles. As shown in Fig. 4F, the binding activity of c‐Jun to the 2 binding sites was evidently increased in the distent colonic smooth muscles compared with the sham controls, indicated by the brighter bands after the incubation with anti‐c‐Jun antibody. The ChIP results were further confirmed by EMSA and super‐shift assays. DNA probes corresponding to the 2 c‐Jun binding sites containing the putative c‐Jun binding sequences and their 5′ and 3′ flanking regions were used. After incubation with the nuclear extracts from the colonic smooth muscles of sham mice, no obvious retarded bands were observed (Fig. 4G). However, incubation of biotin‐labelled probes targeting Site 1 and Site 2 with the nuclear extracts from the distent colonic smooth muscles resulted in clear retarded bands (Fig. 4G). Two hundred‐fold excess of unlabelled probes prevented the labelled probes from binding to the nuclear extracts, which was abrogated by unlabelled mutational probe (Fig. 4G). Furthermore, incubation of anti‐c‐Jun antibody caused a super‐shift band (Fig. 4G), indicating the presence of c‐Jun in the DNA–protein binding complex. These results elucidated that c‐Jun could effectually drive *miR‐34c* transcription in the colonic SMCs *via* directly binding to each binding site within the *miR‐34c* promoter and the ability could be enhanced by the persistent stretch.

### c‐Jun was activated by ERK1/2 signalling in response to stretch

**Figure 5:**
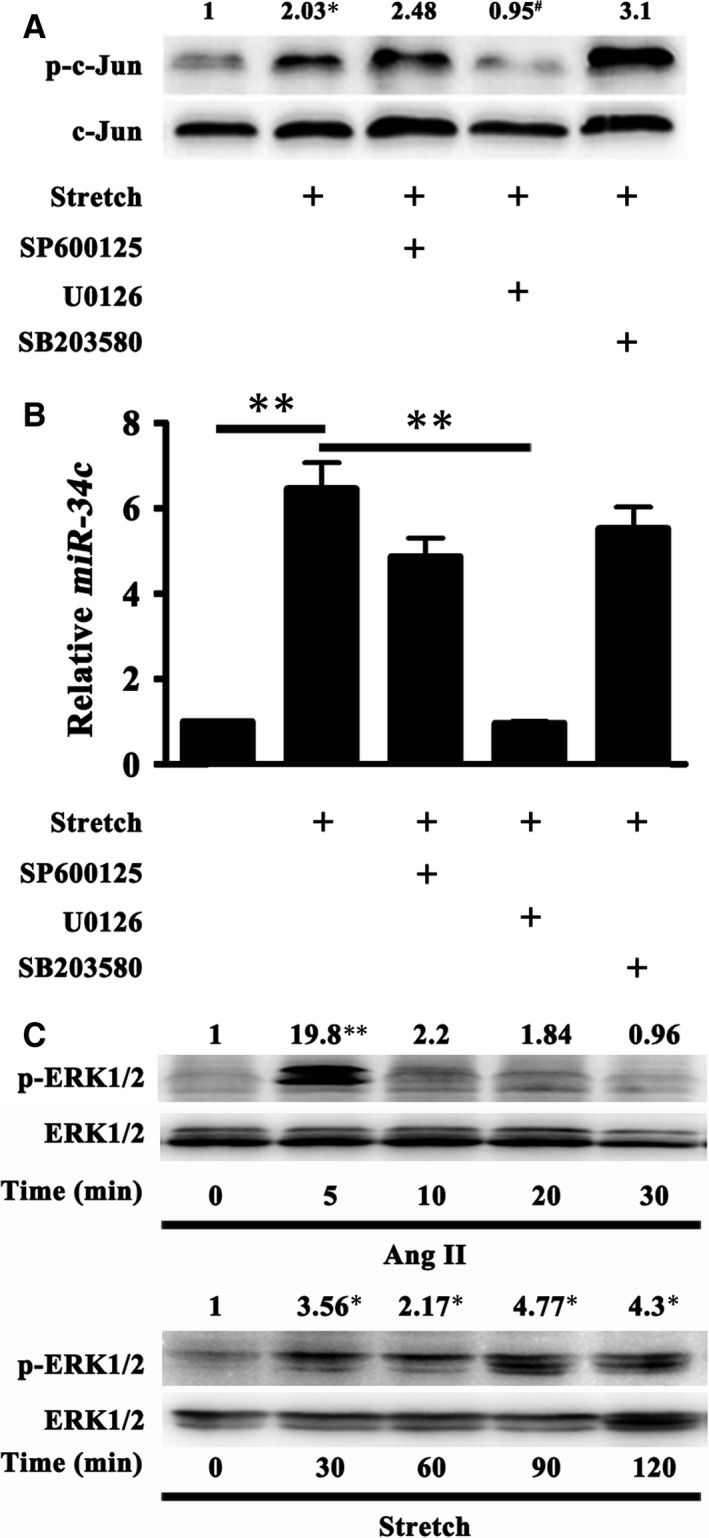
c‐Jun was activated by ERK1/2 signalling in response to stretch. (**A**) Persistent stretch on the cultured mouse colonic SMCs for 12 hrs activated c‐Jun (*n* = 5; *P < 0.05), which, however, was significantly abrogated by the ERK1/2 inhibitor (U0126) (*n* = 5; #*P* < 0.05). But neither the JNK inhibitor (SP600125) nor the p38 inhibitor (SB203580) had such effect. (**B**) Likewise, only U0126 significantly abolished the persistent stretch‐induced increase of *miR‐34c* in colonic SMCs. (*n* = 5; **P < 0.01) (**C**) 100 nM AngII quickly activated ERK1/2 kinase for about 5 min. in the cultured mouse colonic SMCs, whereas the stretch kept ERK1/2 kinase activated within the whole exertion (~120 min.).

MAP kinase pathways are known to be activated in response to a number of stimuli and modulate the activation of transcription factors. In our previous study, we found increased phosphorylation of JNK, ERK1/2 and p38 in the distent colonic smooth muscles of mouse and HD patients 17, but which signalling was implicated in c‐Jun activation was unclear. To clarify the role of individual MAP kinase in the activation of c‐Jun in response to persistent stretch, *in vitro* persistent stretch for 12 hrs on the cultured mouse colonic SMCs was applied following the treatment with JNK inhibitor (SP600125, 20 μM), ERK1/2 inhibitor (U0126, 50 μM) or p38 inhibitor (SB203580, 20 μM). The persistent stretch highly activated c‐Jun and elevated *miR‐34c* expression; however, only U0126 significantly abolished the stretch‐induced activation of c‐Jun and increase of *miR‐34c* (Fig. 5A and B). These data suggested that ERK1/2 kinase was involved in the activation of c‐Jun and consequent up‐regulation of *miR‐34c* in the persistently stretched colonic SMCs. As the activation of ERK1/2 kinase is supposed to be temporary in response to several stimuli such as angiotensin, integrins, we compared the duration of ERK1/2 kinase activation by angiotensin II (AngII) and persistent stretch. As shown in Fig. 5C, 100 nM AngII quickly induced evident activation of ERK1/2 kinase, which lasted no more than 5 min. However, the stretch‐induced ERK1/2 kinase activation was maintained within the whole duration (120 min.), indicating that the persistent stretch was able to keep a sustained activation of ERK1/2 kinase in colonic SMCs.

### Stretch‐induced ERK1/2 activation was Ca^2+^ dependent

**Figure 6:**
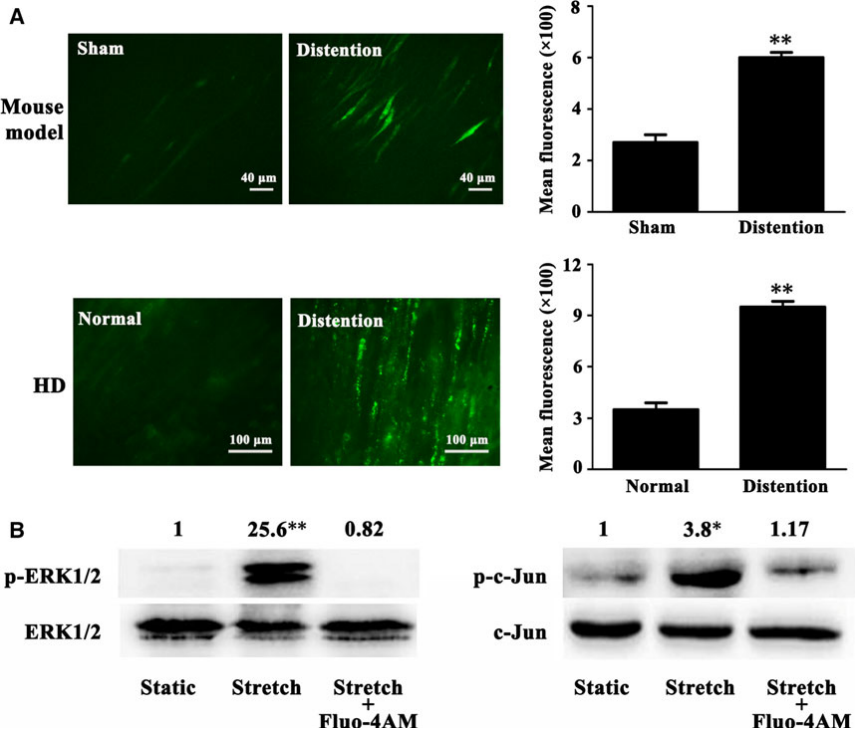
Stretch‐induced ERK1/2 activation was through Ca^2+^ overload. (**A**) The intracellular Fluo‐4AM‐labelled Ca^2+^ fluorescence intensity in the colonic smooth muscles was overtly stronger in the distent colon of the mouse model and HD patients. (*n* = 5; **P < 0.01) (**B**) Persistent stretch‐activated ERK1/2 kinase and downstream c‐Jun in the cultured mouse SMCs, which were evidently abolished by the introduction of Ca^2+^ chelator, Fluo‐4AM. (*n* = 5; *P < 0.05; **P < 0.01).

Influx of Ca^2+^ in SMCs could be one of the first responses to stretch. As an important second messenger, Ca^2+^ transduces multiple extracellular stimuli to activate intracellular signalling pathways. Thus, Ca^2+^ in the colonic smooth muscles disassociated from the mouse model and HD patients was detected in the present study. The intracellular Ca^2+^ fluorescence intensity in the colonic smooth muscles was stronger in the distent colon (*P* < 0.01; Fig. 6A). Notably, pre‐treatment with the intracellular Ca^2+^ chelator, Fluo‐4AM, abrogated the stretch‐induced ERK1/2 activation and following phosphorylation of c‐Jun in the cultured SMCs (Fig. 6B), indicating that the stretch‐activated ERK1/2 signalling was through Ca^2+^ overload.

## Discussion

It is well accepted that loss and/or functional impairment of ICC contribute to GMDs 4, inspiriting us to investigate the cause of ICC changes to better understand the development of GMDs and seek for promising therapies. SCF, mainly produced by the GI smooth muscles and enteric neurons, is a pivotal factor to keep ICC viable and functions by activating the membrane receptor, KIT, of ICC. Unfortunately, no documents have demonstrated the regulation on SCF expression related to GMDs except one suggesting that SCF expression was mediated by decreased endogenous insulin‐like growth factor (IGF)‐1 in diabetic colonic dysmotility 22. Therefore, we had a special interest in how SCF was decreased in GMD progress in the present study. We declared that SCF expression in colonic SMCs was post‐transcriptionally suppressed by increased *miR‐34c*, which lead to decreased ICC and GI dysmotility.

MicroRNA expression could usually be altered by diverse extrinsic and intrinsic factors. A battery of studies documented that stretch provoked significant alteration of microRNA profile in SMCs, resulted in corresponding biological effects. In human aortic SMCs, *miR‐21* was up‐regulated by cyclic stretch, which was implicated in proliferation and apoptosis 23, whereas *miR‐145* was suppressed to alter vascular SMC phenotype 24. In human airway SMCs, stretch up‐regulated *miR‐26a* and induced SMC hypertrophy 25. In fact, the gut SMCs are often exposed to stretch when chyme is delivered along the GI tract. It is reasonably presumed that excessive exposure to stretch could induce microRNA changes capable of diminishing SCF production in the SMCs. In this study, the DGIP mice and HD patients showed remarkable SCF deficiency concomitant with apparent distent colon, suggesting a possible link between SCF reduction and stretch bridged by microRNA alteration. Therefore, we established the chronic colonic distention mouse model to further investigate the persistent stretch‐induced microRNA changes. Through microRNA array, we screened out 37 of ≥twofold up‐regulated microRNAs in smooth muscles of the distent mouse colon, from which *miR‐34c* was picked up because it was supposed to target *SCF* 3′UTR according to bioinformatic algorithm. Knock‐down or overexpression of *miR‐34c* together with luciferase analysis consolidated the negative regulation of *miR‐34c* on SCF expression. The rise of *miR‐34c* was also observed in the DGIP mice and HD patients with distent colon *in vivo*, as well as the cultured mouse SMCs exposed to stretch for 12 hrs *in vitro*. Significantly, the introduction of *miR‐34c* specific inhibitor abolished the stretch‐induced SCF decrease, again, suggesting that the highly expressed *miR‐34c* in the bowel SMCs under the stretched condition was one of the substantial mediators on SCF production. In addition, the regulation of *miR‐34c* on SCF was recently reported in human vascular SMCs and colorectal cancer cells 26, 27, further supporting our results.

Regarding the regulation of *miR‐34c* expression, it was reported that the decreased *miR‐34c* could be attributed to DNA hyper‐methylation in breast tumour‐initiating cells 28. Deletion of Dicer that is required for microRNA maturation in vascular SMCs completely ablated microRNAs 29. Moreover, a few reports showed that AP‐1 could promote microRNA expressions at transcriptional level 30, 31, 32, and AP‐1 could be activated by stretch on vascular and airway SMCs 21, 33. In the present study, we confirmed that in response to persistent stretch, AP‐1/c‐Jun was able to promote *miR‐34c* transcription by binding to either binding site within the *miR‐34c* promoter in mouse colonic SMCs. To our knowledge, it was the first time to reveal the transcriptional regulation of AP‐1/c‐Jun on *miR‐34c*. Apart from AP‐1, some other transcription factors including p53 and E2F1 were competent to facilitate *miR‐34c* transcription 34, 35, but their roles under the state of stretch have not been elucidated.

The stretch‐activated AP‐1 could be through several signalling pathways, among which the MAP kinases were widely studied. For example, stretch‐activated ERK1/2 signalling altered the vascular SMC phenotype 24. JNK signalling activated by stretch promoted proliferative modulation of human bladder SMCs 36. In gastric SMCs, blockage of p38 signalling ameliorates delayed gastric emptying of diabetic rats 37. In the present study, we demonstrated that the stretch‐activated AP‐1/c‐Jun was *via* ERK1/2 but not JNK or p38, which was responsible for the *miR‐34c* up‐regulation. Additionally, our results showed that the activation of ERK1/2 in mouse colonic SMCs was originated from the intracellular Ca^2+^ overload in response to the persistent stretch. Ca^2+^ could come from Ca^2+^ influx by opening plasma membrane stretch activator channel (SAC) as well as Ca^2+^ release from endoplasmic reticulum *via* IP_3_ and ryanodine channels 17, 38.

Generally, the arrested GI peristalsis would lead to prolonged retention time of food that causes bowel distention. Here, we underlined the contribution of colonic distention/stretch to the SCF deficiency and GMD development. Surely, we did not exclude that with the progression of GMDs, the food residue would aggravate the stretch to the GI wall, which, unavoidably, deteriorated GMDs.

**Figure 7:**
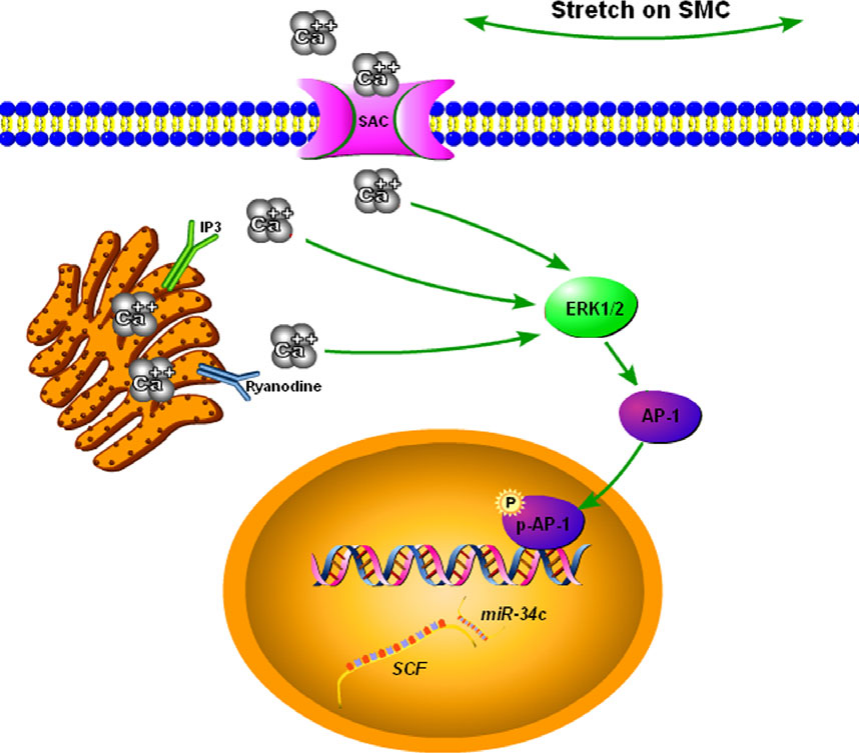
The scheme shows the pathway of SCF reduction in stretched colonic SMCs. The persistent stretch induces Ca^2+^ overload in SMCs, which activated ERK1/2 and ensuing phosphorylation of AP‐1/c‐Jun. The activated AP‐1/c‐Jun promotes *miR‐34c* transcription, resulting in the down‐regulation of its target SCF.

In conclusion, we elucidated that *miR‐34c* was highly increased in the colonic SMCs exposure to persistent stretch, resulting in the down‐regulation of SCF that possibly contributed to the deficiency of ICC. Furthermore, transcriptional factor AP‐1/c‐Jun, activated by Ca^2+^‐provoked ERK1/2 signalling, was involved in the stretch‐enhanced *miR‐34c* transcription (Fig. 7). Our findings provided a comparatively comprehensive pathway, through which the persistent stretch could impede gut motility, and suggested that targeting key molecules within the pathway could represent a promising therapy to prevent or ameliorate GMDs.

## Conflict of interest statement

The authors declare that they have no conflicts of interest.

## Contributors

S. Y, F. D, D. L, H. S, B. W, T. S, Y. W, P. S and F. J performed experiments. S. Y, F. D and D. Z wrote the manuscript. S. Y, F. J and D. Z gave suggestion on discussion and interpretation on the data. D. Z is the guarantor of this work and, as such, had full access to all the data in the study and takes responsibility for the integrity of the data and the accuracy of the data analysis.

## Supporting information


**Figure S1** (**A**) Immunofluorescence staining showed that mouse colonic SMCs were labelled with SCF (red), and most of the cells were infected with the lentivirus expressing EGFP (green). (**B**) Schematic diagram of the *SCF* 3′UTR and the putative seed‐matching sequences (2643–2650 nts in upper case) that are complementary to the *miR‐34c* sequences. The 8 mer seed‐matching sequence was mutated (in grey).

**Figure S2** (**A**) *miR‐34c* level was elevated by 100 nM TPA in a time‐dependent manner. (*n* = 5, *P < 0.05) (**B**) Within the *miR‐34c* promoter (in upper case), there are 2 putative binding sites of c‐Jun at Site 1 (−405~−395 bp) and Site 2 (−371~−361 bp) indicated by circled numbers. Lower‐case letters indicates the sequence of *pre‐miR‐34c*.

**Table S1** Antibodies
**Table S2** Probes used in EMSA
**Table S3** Primers used in ChIP
