# Supplementary figures and images for: Persistent distention of colon damages interstitial cells of Cajal through Ca2+‐ERK‐AP‐1‐miR‐34c‐SCF deregulation
Source: J Cell Mol Med. 2017 Jun 4;21(9):1881–92. doi: 10.1111/jcmm.13108 (PMC5571545; doi:10.1111/jcmm.13108)

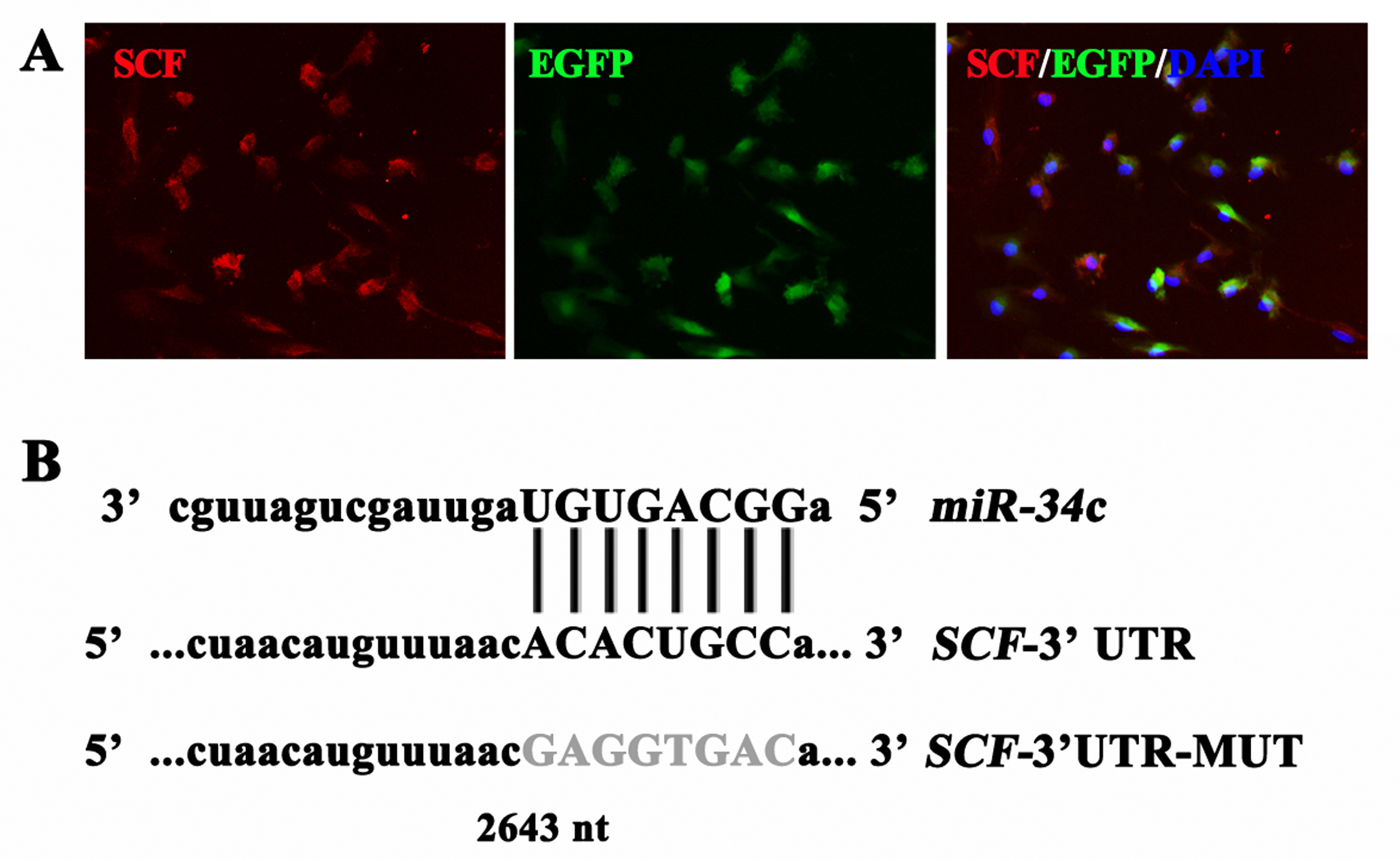

Supplement: Supplementary file 1 — Figure S1 (A) Immunofluorescence staining showed that mouse colonic SMCs were labelled with SCF (red), and most of the cells were infected with the lentivirus expressing EGFP (green). (B) Schematic diagram of the SCF 3′UTR and the putative seed‐matching sequences (2643–2650 nts in upper case) that are complementary to the miR‐34c sequences. The 8 mer seed‐matching sequence was mutated (in grey). [file JCMM-21-1881-s001.tif]

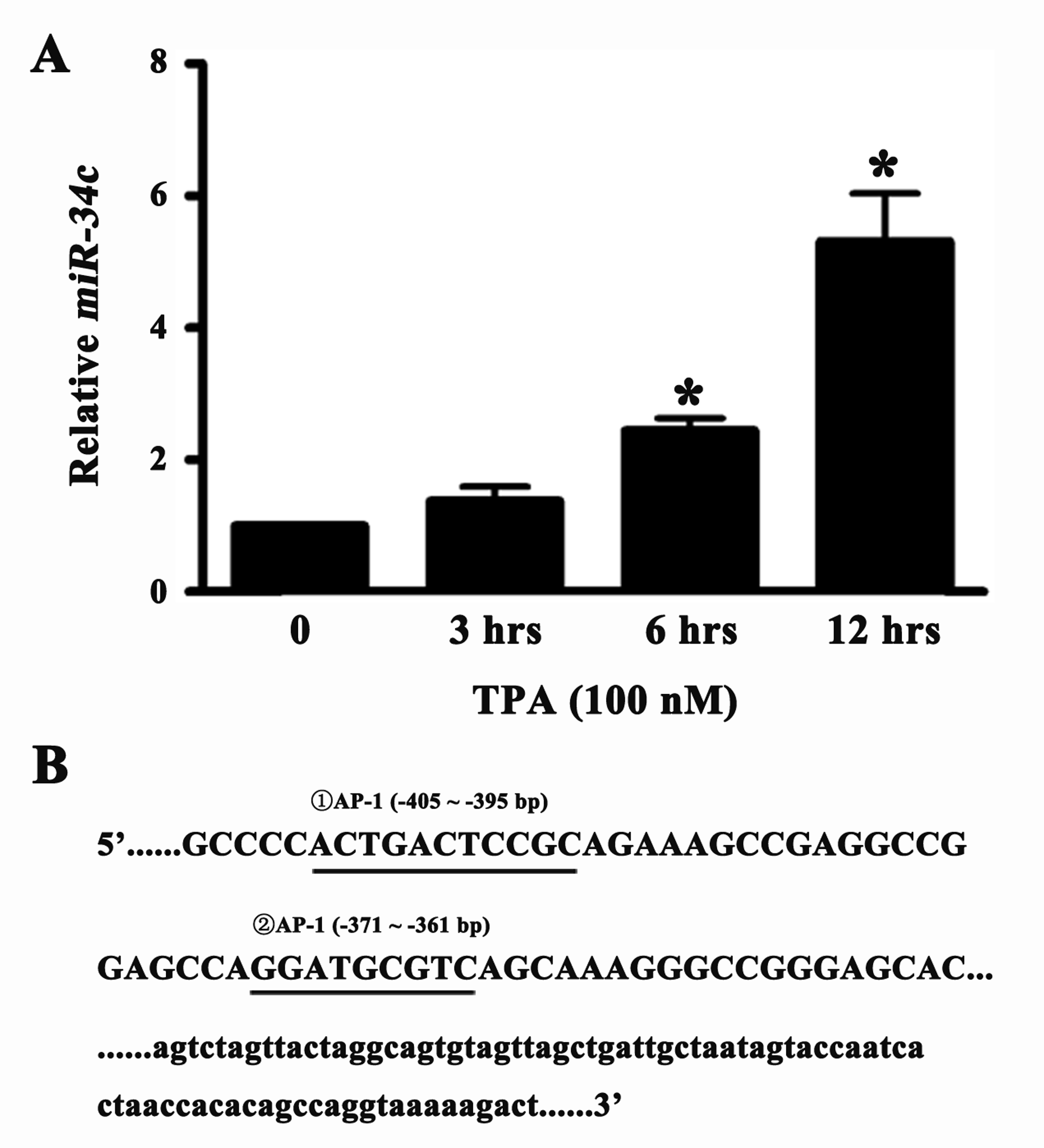

Supplement: Supplementary file 2 — Figure S2 (A) miR‐34c level was elevated by 100 nM TPA in a time‐dependent manner. (n = 5, *P < 0.05) (B) Within the miR‐34c promoter (in upper case), there are 2 putative binding sites of c‐Jun at Site 1 (−405~−395 bp) and Site 2 (−371~−361 bp) indicated by circled numbers. Lower‐case letters indicates the sequence of pre‐miR‐34c. [file JCMM-21-1881-s002.tif]
