# Supplementary material for: Persistent distention of colon damages interstitial cells of Cajal through Ca2+‐ERK‐AP‐1‐miR‐34c‐SCF deregulation
Source: J Cell Mol Med. 2017 Jun 4;21(9):1881–92. doi: 10.1111/jcmm.13108 (PMC5571545; doi:10.1111/jcmm.13108)
Supplement: Supplementary file 3 — Table S1 Antibodies Table S2 Probes used in EMSA Table S3 Primers used in ChIP [file JCMM-21-1881-s003.doc]

| Table S1 Antibodies | | | |
| --- | --- | --- | --- |
| Antibody | Supplier | Western blot | Immunofluorescensce staining |
| mouse anti-SCF | Santa Cruz, USA | 1:1000 |  |
| rat anti-KIT | eBiosciecnce, USA | 1:800 | 1:200 |
| rabbit anti-c-Jun | Cell Signaling Technology, USA | 1:1000 | 1:200 |
| rabbit anti-p-c-Jun | Cell Signaling Technology, USA | 1:1000 | 1:200 |
| rabbit anti-ERK1/2 | Cell Signaling Technology, USA | 1:2000 |  |
| rabbit anti-p-ERK1/2 | Cell Signaling Technology, USA | 1:1000 |  |
| mouse anti-β-actin | Santa Cruz, USA | 1:5000 |  |
| HRP-conjugated goat anti-mouse IgG | Santa Cruz, USA | 1:4000 |  |
| HRP-conjugated goat anti-rabbit IgG | Abcam, USA | 1:2000 |  |
| Cy3-conjugated goat anti-rat IgG | Life Technologies, USA |  | 1:400 |

| Table S2 Probes used in EMSA | | |
| --- | --- | --- |
| Binding site in *miR-34c* promoter |  | Sequence (5’ to 3’) |
| c-Jun-1 | Forward | CGCTGCAGCCCCACTGACTCCGCAGAAAGCCGAGG |
|  | Reverse | CCTCGGCTTTCTGCGGAGTCAGTGGGGCTGCAGCG |
| c-Jun-1-MUT | Forward | CGCTGCAGCCCC*GTGAGTGTAAT*AGAAAGCCGAGG |
|  | Reverse | CCTCGGCTTTCT*ATTACACTCAC*GGGGCTGCAGCG |
| c-Jun-2 | Forward | GCCGGAGCCACGATGCGTCAGCAAAGGGCCGGG |
|  | Reverse | CCCGGCCCTTTGCTGACGCATCGTGGCTCCGGC |
| c-Jun-2-MUT | Forward | GCCGGAGCCACG*GCGTGCCGGTG*AAGGGCCGGG |
|  | Reverse | CCCGGCCCTT*CACCGGCACGC*CGTGGCTCCGGC |
| Nucleotides in italic are mutated. | | |

| Table S3 Primers used in ChIP | | |
| --- | --- | --- |
| Binding site in *miR-34c* promoter |  | Sequence (5’ to 3’) |
| c-Jun-1 | Forward | ACCGTTCGGCTACCTGTG |
|  | Reverse | GTCGCTGCCTTGGTGATA |
| c-Jun-2 | Forward | GACAATCACTAACTCCACTGCCATC |
|  | Reverse | GCTCTTACACACAGGTAGCCGAACG |
| GAPDH | Forward | CCTCTGCGCCCTTGAGCTAGGA |
|  | Reverse | CACAAGAAGATGCGGCCGTCTC |
